# Moschus ameliorates glutamate-induced cellular damage by regulating autophagy and apoptosis pathway

**DOI:** 10.1038/s41598-023-45878-7

**Published:** 2023-10-30

**Authors:** Danni Xie, Caiyou Song, Tao Qin, Zhenwei Zhai, Jie Cai, Jingyi Dai, Tao Sun, Ying Xu

**Affiliations:** 1 State Key Laboratory of Southwestern Chinese Medicine Resources, School of Pharmacy, Chengdu University of Traditional Chinese Medicine, Chengdu, 611137 China; 2 School of Medical Information Engineering, Chengdu University of Traditional Chinese Medicine, Chengdu, 611137 China; 3 Hospital of Chengdu University of Traditional Chinese Medicine, Chengdu, 610072 China

**Keywords:** Cell biology, Drug discovery

## Abstract

Alzheimer's disease (AD), a neurodegenerative disorder, causes short-term memory and cognition declines. It is estimated that one in three elderly people die from AD or other dementias. Chinese herbal medicine as a potential drug for treating AD has gained growing interest from many researchers. Moschus, a rare and valuable traditional Chinese animal medicine, was originally documented in *Shennong Ben Cao Jing* and recognized for its properties of reviving consciousness/resuscitation. Additionally, Moschus has the efficacy of “regulation of menstruation with blood activation, relief of swelling and pain” and is used for treating unconsciousness, stroke, coma, and cerebrovascular diseases. However, it is uncertain whether Moschus has any protective effect on AD patients. We explored whether Moschus could protect glutamate (Glu)-induced PC12 cells from cellular injury and preliminarily explored their related action mechanisms. The chemical compounds of Moschus were analyzed and identified by GC–MS. The Glu-induced differentiated PC12 cell model was thought to be the common AD cellular model. The study aims to preliminarily investigate the intervention effect of Moschus on Glu-induced PC12 cell damage as well as their related action mechanisms. Cell viability, lactate dehydrogenase (LDH), mitochondrial reactive oxygen species, mitochondrial membrane potential (MMP), cell apoptosis, autophagic vacuoles, autolysosomes or autophagosomes, proteins related to apoptosis, and the proteins related to autophagy were examined and analyzed. Seventeen active compounds of the Moschus sample were identified based on GC–MS analysis. In comparison to the control group, Glu stimulation increased cell viability loss, LDH release, mitochondrial damage, loss of MMP, apoptosis rate, and the number of cells containing autophagic vacuoles, and autolysosomes or autophagosomes, while these results were decreased after the pretreatment with Moschus and 3-methyladenine (3-MA). Furthermore, Glu stimulation significantly increased cleaved caspase-3, Beclin1, and LC3II protein expression, and reduced B-cell lymphoma 2/BAX ratio and p62 protein expression, but these results were reversed after pretreatment of Moschus and 3-MA. Moschus has protective activity in Glu-induced PC12 cell injury, and the potential mechanism might involve the regulation of autophagy and apoptosis. Our study may promote research on Moschus in the field of neurodegenerative diseases, and Moschus may be considered as a potential therapeutic agent for AD.

## Introduction

Alzheimer's disease (AD) is a progressive disease that initiates with mild memory loss and may progress to full impairment of cognitive and executive functioning^1^. One in three elderly people dies from AD or another form of dementia^2,3^. Compared with the number of people who died from AD in the last five years, the number in 2020 increased by 15,925, which was 13% higher than expected^2,3^. By 2025, it is predicted to reach 7.2 million people aged 65 or older suffering from AD, up by 11% from 6.5 million in 2022, and the number is expected to grow to 13.8 million by 2060^4^. Furthermore, individuals with AD spend 12% of their income on out-of-pocket healthcare services compared with 7% for individuals without AD^5^. Therefore, developing effective treatment strategies for AD will ameliorate the financial burden of healthcare for patients.

Hydrogen peroxide (H_2_O_2_), Amyloid β (Aβ), and Glutamate (Glu) are considered as common damaging agents in AD models^6^. H_2_O_2_ is considered as a classic inducer of oxidative stress^7^ because it is an endogenously produced reactive oxygen species (ROS), which serves as a precursor of hydroxyl radicals and has a signaling capacity.^8^. Aβ oligomers exert neurotoxicity via inducing caspase-3 mediated apoptosis^9^. Furthermore, the neurotoxicity of Aβ aggregation involves diverse cellular and molecular mechanisms, such as generating ROS and inducing apoptosis^10,11^. Moreover, Glu is one of the primary excitatory neurotransmitters in the central nervous system, and excitatory neurotoxicity could emerge when Glu levels are significantly enhanced^12^. There is mounting evidence that Glu excitotoxicity plays a role in slow-evolving neurodegeneration^13,14^. Glu excitotoxicity is thought to be the final common pathway of neuronal injury for some degenerative neural disorders, such as AD^15^, AIDS dementia^16^, Amyotrophic Lateral Sclerosis^17^, and Parkinson's Disease^18^. Furthermore, earlier studies provided evidence that Glu-induced neurotoxicity is one of the most critical factors leading to the loss of neurons in AD^19^. Normal levels of Glu play an essential role in learning and memory^20^, while abnormally high Glu levels could lead to over-excitation of the nerve cell, causing cell damage and/or death^21^. In addition, Glu-induced cytotoxicity is associated with autophagic cell death, and the inhibited autophagy attenuates Glu-induced neuronal death^22,23^. The Glu receptor-mediated excitotoxicity occurs through activating Glu receptors (NMDA receptors), which causes a massive influx of Ca^2+^ and cell death^13,24^. These downstream effects following large Ca^2+^ influx include mitochondrial membrane depolarization, caspase activation, toxic oxygen, and nitrogen free radicals’ production, and cellular toxicity^25,26^. Meanwhile, excessive uptake of calcium or generation of ROS induces activation of the mitochondrial permeability transition and subsequent release of calcium and pro-apoptotic factors into the cytosol^27,28^. It has been reported that the activation of autophagy is involved in Glu-induced neuronal injury, and the ratio of LC3II/LC3I increases^29^. Furthermore, Glu was regarded as a common damage agent in the apoptotic model of differentiated PC12 cells^30^. It is well known that Glu can induce cytotoxicity against PC12 cells^31^. The study found that the toxicity of Glu (0, 5, 10, 15, 20 mM) to PC12 cells occurred dose-dependent, and IC50 appeared at 20 mM Glu for 24 h^32^. These studies lay the foundation for using Glu for cytotoxicity in AD in vitro models.

After induced by nerve growth factor (NGF), differentiated PC12 cells are close to neurons in terms of morphology, physiological and biochemical functions^10^, and also synthesize acetylcholine (Ach)^11^. Ach is the neurotransmitter used by all cholinergic neurons^12^ and is closely involved in learning and memory processes^13^. Furthermore, the degeneration of cholinergic neurons is a prominent feature of AD^14^. The classical “cholinergic hypothesis” posits that AD results from the diminished synthesis of the vital neurotransmitter acetylcholine^15^. Additionally, PC12 cells could express the NGF receptors tyrosine kinase (Trk) A and p75 neurotrophin receptor (p75^NTR^)^33^. TrkA and p75^NTR^ are closely associated with basal cholinergic forebrain (BCF) neuron survival, and dysfunction of BCF neurons is a characteristic of AD^34^.

Volatile essential oils from plant or animal sources were employed to ameliorate emotional changes of different neurodegenerative diseases, particularly for treating dementia in both experimental animal models and humans^35–37^. Moschus, as one of the rarest and most valuable animal medicines in traditional Chinese medicines, is derived from dry secretions of mature male musk deer, including *Moschus berezovskii* Flerov, *Moschus sifanicus* Przewalski, and *Moschus moschiferus* Linnaeus (documented in Chinese Pharmacopoeia (2020 edition))^38^. Moschus was originally documented in *Shennong Ben Cao Jing* and had a history as a medicinal material usage for almost two millennia^39^. Moschus is taken as a regular refreshing and resuscitating drug for treating pulmonary heat and unconsciousness, stroke and phlegm syncope, neurasthenia, convulsions, bruising, and sore throat^38,40,41^. The relative modern pharmacology of Moschus suggests that it has efficacy in neurological action, cardio-cerebrovascular action, anti-oxidant, anti-apoptotic, anti-inflammatory, and immune action^42,43^. Moschus exerts a dual regulatory effect on blood–brain barrier (BBB) permeability. Under physiological conditions, Moschus increases the concentration of drugs in brain tissue, while under pathological states, Moschus reduces BBB permeability as well as protects the structural integrity of the BBB^44,45^. In comparison to the model group, BBB permeability and Evans blue (EB) content of the brain could be decreased by gavage administration with 0.0666 g/kg Moschus in cerebral ischemia–reperfusion injury (CIRI) mice^46^. Meanwhile, 0.025 and 0.05 g/kg Moschus effectively alleviated neurological deficits by gavage administration in CIRI rats compared with the model group^47^. Moschus combined with Borneolum Synthcticum harbors neuroprotective benefits against ischemic stroke through reducing the volume of cerebral infarction and regulating the expression of apoptosis-related proteins^48^. As compared to Chronic Unpredictable Mild Stress model mice, the Moschus-treated group showed an increase in serum catalase levels, glutathione peroxidase levels, and superoxide dismutase levels as well as a reduction in the duration of activity^49^. Moschus at 4, 8, and 16 μg/ml doses significantly protected PC12 cells from Na_2_S_2_O_4_-induced damage^50^. In clinical practice, Moschus, as the main medicinal material, is commonly used in prescriptions for treating ischemic strokes, such as Angong Niuhuang Pill (ANP)^51^, Tongqiao Huoxue Decoction (THD)^52^, and Xingnaojing Injection (XNJI)^53^. In comparison to the model group, EB content of cerebral cortex and brain water content have a reduced trend after ANP-treated closed brain injury rats^54^. The previous study found that the serum containing THD exerts protective effects on cell proliferation and membrane permeability in Glu-induced PC12 cells^55^. Compared with the group treated with vehicle, LC3II protein expression was downregulated, and p62 protein expression was upregulated after XNJI treatment in middle cerebral ischemia-reperfusion rats and PC12 cells of serum-free condition^56^. These researches will lay credence for the application of Moschus in AD. However, the neuroprotective effect and the underlying neuroprotective mechanism of Moschus on Glu-injured PC12 cells remain unclear. Thus, our study aims to explore the efficacy and potential mechanism of Moschus in AD cellular model. The application of traditional drugs could lay a foundation for exploring candidate drugs to prevent and treat AD.

Cell death is classified as accidental cell death and programmed cell death (PCD)^57^. The two primary categories of programmed cell death are apoptosis and autophagic cell death^58^. Apoptosis, as one of the most common forms of PCD, is mediated by caspase proteases^59^. The antiapoptotic B-cell lymphoma 2 (Bcl-2) protein and the proapoptotic Bax protein are considered as essential effectors of apoptosis^60^. During apoptosis, cleaved caspase-3 is a well-accepted biomarker for apoptosis-induced cell death^61^. Autophagy is taken as the process by intracellular constituents for degradation in the lysosome^62,63^, which allows the cell to adapt to changing environmental conditions and eliminates misfolded or aggregated proteins and damaged organelles^64^. Autophagy is essential in neuronal homeostasis, and its dysfunction is directly related to neurodegenerative diseases^65–67^. Research has shown that autophagy has been proven to protect cells from apoptosis^68^. The impairment of autophagy leads to an increase in neuronal apoptosis^69^. Beclin 1, SQSTM1/p62 (sequestosome 1), and LC3 are markers of autophagy^70,71^. Beclin 1 is an essential protein for initiating autophagy^72^. The presence of a putative BH3-like domain in Beclin 1 is thought to mediate the interaction of Beclin 1 with anti-apoptotic Bcl-2 family proteins, which may be inhibited by BH3-only proteins^73^. The BH3-only proteins, which constitute a subfamily of Bcl-2, play a regulatory role by inhibiting the anti-apoptotic Bcl-2 proteins and activating Bax and Bak, thereby inducing mitochondrial outer membrane permeabilization^74^. Bcl-2 and Bcl-xL, as other constituents of the Bcl-2 family, exert anti-apoptotic effects by either directly interacting with the effector proteins BAK and BAX or sequestering pro-apoptotic BH3-only members^75^. When autophagy is inhibited, the expression of Beclin 1 decreases, while the expression of p62 increases^76,77^; at the same time, the conversion of LC3I to LC3II through binding to autophagosome membrane was blocked, and the ratio of LC3II/LC3I was decreased^78,79^. 3-Methyladenine (3-MA) was commonly used as an autophagy inhibitor^80^, preventing autophagy via inhibiting autophagosome formation at an early stage^81^. It was found that 3-MA could protect from apoptosis in PC12 cells following serum deprivation^82^.

The PC12 cells were selected as the AD cellular model to investigate whether Moschus possesses the protective capacity against Glu-induced cell injury and preliminarily explore their related action mechanisms.

## Materials and methods

### Materials

Sichuan Fengchuntang Traditional Chinese Medicine Co., Ltd: Moschus; Chinese Academy of Sciences Type Culture Collection Committee: highly differentiated PC12 cells; Gibco Co.: RPMI-1640 medium (C11875500BT) and fetal bovine serum (FBS) (10099141); Sigma-Aldrich: Glu (56-86-0); Hyclone Co.: Penicillin and Streptomycin (PYG0016); Dojindo Molecular Technologies: cell counting kit-8 (CCK-8) (CK04); BOSTER Biological Technology Co. Ltd: JC-1 assay kit (J6004L) and MitoSOX Red (HY-D1055); MCE: dansylcadaverine (MDC) (HY-D1027) and 3-MA (HY-19312); Suzhou Yuheng Biotechnology Co., Ltd: YF®488-Phalloidin YF®488 (YP0059); Jiancheng Bioengineering Institute: Lactate dehydrogenase (LDH) (A020-2-2) and bicinchoninic acid (BCA) (W041-1-1); Wuhan Sanying Biotechnology Co. Ltd: Bax (50599-2-lg), Bcl-2 (26593-1-AP), GAPDH (60004-1-Ig); Abmart: cleaved caspases-3 (TA7022); Bioss: Beclin 1 (bs-1353R) and SQSTM1/p62 (bs-55207R); Immunoway: LC3B (YT7938).

### The sample treatment

Moschus was precisely weighed to 50 mg in a 5 ml measuring flask and diluted with absolute ethanol to the mark, shaken, and diluted to a 10 mg/ml standard solution. A 0.22 μm membrane filtered the supernatant and then analyzed by GC–MS. The ingredients of the sample were performed with the NIST14.L GC–MS library.

### GC–MS analysis

GC conditions were as follows: the carrier gas was helium at a flow rate of 1 mL/min; the analysis of Moschus was performed using a temperature program consisting of an initial hold at 60 °C, ramped up to 150 °C at a rate of 10 °C/min and held at 150 °C for 5 min, then ramped to 280 °C at a rate of 5 °C/min and saved at 280 °C for 5 min, with the split ratio of 10:1; the injection port temperature was set at 280 °C and introduced via the auto-injector using an injection volume of 1 µL.

MS conditions were as follows: ion source temperature, 230 °C; quadrupole temperatures, 150 °C; full scan mode.

### Cell culture

Highly differentiated PC12 cells were growing and cultured in RPMI-1640 medium containing 10% FBS and antibiotics (100 μg/ml Streptomycin and 100 U/ml Penicillin) at an incubator with a 5% CO_2_ atmosphere at 37 °C. PC12 cells could be passaged or seed-plated by the time 80–90% confluence is reached.

### The drug treatment and cell viability

Cell viability was commonly measured by the CCK-8 kit^83^. PC12 cells were treated with different concentrations of Moschus (0.05, 0.1, 0.2, 0.3, and 0.4 mg/ml) and Glu (5, 10, 15, 20, 30, and 40 mM) for 24 h, respectively. Based on the concentration screening of Moschus and Glu, PC12 cells were pretreated with Moschus (0.05 and 0.1 mg/ml) and 3-MA (5 mM)^84^ for 24 h, then incubated with Glu (20 mM) for an additional 24 h, then incubated at an incubator for 2 h after adding 100 μl CCK-8 solution to each well. After incubation, the absorbance was evaluated by a Multiskan FC microplate reader (Thermo Scientific™, Waltham, MA, USA) at 450 nm, then recorded the OD. The cell viability was calculated according to the CCK-8 kit instructions.

### LDH assay

PC12 cells were subsequently pretreated with Moschus (0.05 and 0.1 mg/ml) and 3-MA (5 mM) for 24 h, then incubated with Glu (20 mM) for an additional 24 h. To assess the cytotoxicity, LDH release in the supernatant was detected using the LDH assay kit according to the manufacturer's instructions.

### Apoptosis assay

Phosphatidyl serine (PS), as a cell membrane phospholipid, is located on the cytoplasmic surface of the membrane of normal viable cells^85^, while PS usually transfers to the outer membrane when apoptotic stress occurs^86^. During necrosis, PS is also translocated to the surface of the plasma membrane^87^. As a phospholipid-binding protein, Annexin V possesses a high affinity for PS^88^, which is considered as an early cellular apoptotic marker. Living cells stained with fluorochrome-tagged Annexin V and PI, have minimal Annexin V fluorescence and minimal PI fluorescence^89^. At the early stages of apoptosis, cells stain brightly with Annexin V but still exclude PI, while they stain brightly with both Annexin V and PI at the late stages of apoptosis^90^. It is also possible for cells with severely damaged membranes and apoptotic cells to stain rapidly and strongly with PI, but not with Annexin V^89^. The FITC-Annexin V/PI Apoptosis kit carried out cell apoptosis according to the manufacturer's instructions. PC12 cells were pretreated with Moschus (0.05 and 0.1 mg/ml) and 3-MA (5 mM)^84^ for 24 h, then incubated with Glu (20 mM) for an additional 24 h. After treatment, the collected cells were stained with 5 μl Annexin V-FITC for 5 min, then stained with 10 μl 20 ug/ml PI and 400 μl PBS to analyzed by flow cytometer (FACSCanto II, BD Company, New York, NY, USA) and laser confocal microscopy (Leica, SP8 SR, Wetzlar, Germany).

### Detection of mitochondrial membrane potential (MMP)

The mitochondrion is regarded as the bioenergetic center of the cell and is also vital to the intrinsic apoptotic pathway^91^. The decrease in MMP is regarded as a hallmark of incipient cell apoptosis^92^. The changes in MMP were detected using a membrane-potential-sensitive probe JC-1^93^. When MMP is high, the cationic JC-1 dye accumulates in the mitochondria matrix to form a J-aggregate that emits red fluorescence, whereas when MMP is low, the monomer emits green fluorescence^94^. A transition of JC-1 from red to green fluorescence indicated the reduction of MMP^95^ and was regarded as a detection index of incipient cell apoptosis. PC12 cells were pretreated with Moschus (0.05 and 0.1 mg/ml) and 3-MA (5 mM) for 24 h, then incubated with Glu (20 mM) for an additional 24 h. According to the manufacturer's instructions, the collected cells were stained with 0.5 mL JC1 dye for 15 min in a cell incubator at 37 ℃. The fluorescence of stained cells was analyzed by a flow cytometer (FACSCanto II, BD Company, New York, NY, USA).

### Mitochondrial ROS measurement

Mitochondria are regarded as the primary site of ROS production and are vulnerable to ROS-induced damage^96^. The increased ROS was associated with decreased MMP and subsequent induction of cell apoptosis^97^. MitoSOX Red is rapidly and selectively targeted to mitochondria, and is oxidized by ROS within mitochondria, emitting red fluorescence^98^. MitoSOX Red was used to detect mitochondrial ROS levels^99^. PC12 cells were pretreated with Moschus (0.05 and 0.1 mg/ml) and 3-MA (5 mM) for 24 h, then incubated with Glu (20 mM) for an additional 24 h. According to the manufacturer’s instructions, the medium was removed, and the cells were digested with EDTA-free trypsin, centrifuged to discard the supernatant, added PBS to wash twice, each time for 5 min; then added 1 mL MitoSOX Red working solution, and incubated at room temperature for 30 min, centrifuged at 400 g for 4 min at 4 °C and discarded the supernatant, subsequently added PBS to wash the cells twice, 5 min each time, finally detected by flow cytometry (FACSCanto II, BD Company, New York, NY, USA) after the cells were resuspended in 1 mL PBS.

### MDC staining assay

MDC is an autofluorescent compound used for labeling autophagic vacuoles^100^. PC12 cells were pretreated with Moschus (0.05 and 0.1 mg/ml) and 3-MA (5 mM) for 24 h, then incubated with Glu (20 mM) for an additional 24 h. According to the manufacturer's instructions, the treated cells were incubated with 50 mM MDC at 37 ℃ for 15 min and washed with PBS three times at 5 min intervals. The fluorescence of cells was observed using laser confocal microscopy (Leica, SP8 SR, Wetzlar, Germany).

### Transmission electron microscope (TEM)

PC12 cells were pretreated with Moschus (0.05 and 0.1 mg/ml) and 3-MA (5 mM) for 24 h, then incubated with Glu (20 mM) for an additional 24 h. Followed by discarding the supernatant, then fixed with EM fixation buffer for 2–4 h at 4 °C, centrifuged to observe cell clumps in the bottom of the tube, followed by coating with 1% agarose. After washing with 0.1 M phosphate buffer PB (pH7.4) three times for 15 min each, the samples were fixed with 1% osmic acid/0.1 M phosphate buffer PB (pH7.4) for 2 h at 20 °C, then washed with 0.1 M phosphate buffer PB (pH7.4) three times for 15 min each. Subsequently, samples were dehydrated in 50%-70%-80%-90%-95%-100%-100% ethanol-100% acetone-100% acetone for 15 min each, followed by treated with acetone: 812 embedding agents (1:1) for 2–4 h, acetone: 812 embedding agents (1:2) at infiltration overnight, and pure 812 embedding agents for 5–8 h. Then the sample was inserted into the embedding plate overnight in the oven at 37 °C after pure 812 embedding agents were poured into the embedding plate and polymerized at 60 °C for 48 h. Subsequently, 60 to 80 nm ultrathin sections were cut using an ultramicrotome with a diamond knife (Leica UC7). Finally, the cells were stained with uranium-lead double staining (2% uranyl acetate saturated alcohol solution, plumbum citrate, dyed for 15 min respectively), then dried at room temperature overnight, subsequently photographed and observed with a TEM (HT7700, Hitachi, Tokyo, Japan).

### Western blotting analyses

PC12 cells were pretreated with Moschus (0.05 and 0.1 mg/ml) and 3-MA (5 mM) for 24 h, then incubated with Glu (20 mM) for an additional 24 h, subsequently lysed by lysis buffer, then collected and centrifuged to obtain the total cell protein. The protein concentration was quantified using a BCA kit. 10% SDS–polyacrylamide gels were adopted for protein separation, and PVDF membranes were taken for protein transfer. After blocking for 1 h with 5% non-fat milk, PVDF membranes are overnight incubated with cleaved caspase-3 (1:1000), Bax (1:1000), Bcl-2 (1:1000), Beclin 1 (1:1000), LC3 (1:1000), and SQSTM1/p62 (1:1000). After three washes with PBS, PVDF membranes are incubated with secondary antibodies linked to HRP for 1 h. The ECL method was applied to visualize the bands. Image J was taken to quantify the levels of protein expression.

### Immunofluorescence

PC12 cells were pretreated with Moschus (0.05 and 0.1 mg/ml) and 3-MA (5 mM) for 24 h, then incubated with Glu (20 mM) for an additional 24 h, subsequently fixated with 4% buffered paraformaldehyde for 20 min, and blocked with 0.2% Triton-X and 1% BSA for 1 h, finally incubated overnight with primary antibodies against LC3. After washing three times with PBS, cells are dyed separately with Cy3-conjugated anti-rabbit IgG for 1 h, 4-6-diamidino-2-phenylindole is added to the confocal dish for 20 min, then visualized by using a laser confocal microscopy (Leica, SP8 SR, Wetzlar, Germany).

### Statistical analysis

The statistical significance was analyzed using one-way ANOVA with LSD or Dunnett’s T3 by SPSS 21.0 software. Graphing was performed using GraphPad Prism 7. Data were shown as mean ± standard error of the mean from at least three independent experiments. Significance levels were given as follows: ***p < 0.001; **p < 0.01; *p < 0.05.

## Results

### Moschus analyzed by GC–MS assay

The chemical components in the Moschus samples were determined by GC–MS spectrometry and searched in the NIST standard library. The chromatographic peaks with a matching degree higher than 80 are selected and referred to the relevant literature^101–108^ to confirm their chemical composition further. 17 chemical ingredients were identified by GC–MS analysis, the result is shown in Table 1.

**Table 1:** The chemical ingredients of the sample Moschus solutions were analyzed by GC–MS.

Peak	RT (min)	Compound	Molecular formula	CAS number	Relative molecular mass	Similarity
1	9.645	Phenol, 4-methyl-	C_7_H_8_O	106-44-5	108	87
2	10.975	1-Penten-3-ol, 3-methyl-	C_6_H_12_O	918-85-4	100	87
3	11.385	Benzoic acid	C_7_H_6_O_2_	65-85-0	122	93
4	12.76	Benzeneacetic acid	C_8_H_8_O_2_	103-82-2	136	93
5	21.695	Cyclopentadecanone	C_15_H_28_O	502-72-7	224	85
6	24.675	5-Cyclohexadecen-1-one	C_16_H_28_O	37609-25-9	236	85
7	26.915	Muscone	C_16_H_30_O	541-91-3	238	88
8	29.455	n-Hexadecanoic acid	C_16_H_32_O_2_	57-10-3	256	92
9	31.595	9-Octadecenal, (Z)-	C_18_H_34_O	2423-10-1	266	85
10	33.04	Oleic Acid	C_18_H_34_O_2_	112-80-1	282	92
11	33.475	Octadecanoic acid	C_18_H_36_O_2_	57-11-4	284	88
12	35.945	Prasterone-3-sulfate	C_19_H_28_O_5_S	651-48-9	368	91
13	36.905	9-Octadecenamide, (Z)-	C_18_H_35_NO	301-02-0	281	93
14	37.69	Phenol, 2,2′-methylenebis[6-(1,1-dimethylethyl)-4-methyl-	C_23_H_32_O_2_	119-47-1	340	88
15	39.26	Androstan-17-one, 3-hydroxy-, (3α,5β)-	C_19_H_30_O_2_	53-42-9	290	87
16	39.73	Androstane-3,17-dione, (5β)-	C_19_H_28_O2	1229-12-5	288	83
17	44.92	Cholesta-3,5-diene	C_27_H_44_	747-90-0	368	84

### Effects of different concentrations of Moschus and Glu on cell viability

**Figure 1:**
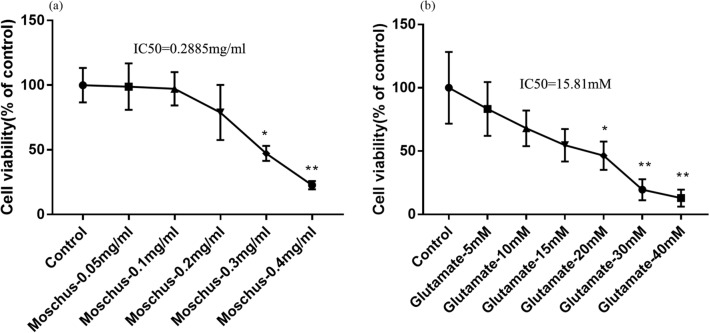
The effect of Moschus and Glu on cell viability in PC12 cells. (**a**) The effect of different concentrations of Moschus on cell viability in PC12 cells. (**b**) The effect of different concentrations of Glu on cell viability in PC12 cells. *p < 0.05 and **p < 0.01 versus the control group.

The screening drug concentrations of Moschus and Glu were evaluated by using the CCK-8 kit. According to the CCK-8 results and IC50 value, Fig. 1a indicated that the IC50 value of Moschus is 0.2885 mg/ml; the cell viability in 0.05 mg/ml and 0.1 mg/ml Moschus had no significant difference, while the cell viability in 0.2 mg/ml, 0.3 mg/ml, and 0.4 mg/ml Moschus was significantly decreased, especially of 0.3 mg/ml and 0.4 mg/ml Moschus. Therefore, the appropriate concentrations of Moschus were 0.05 mg/ml and 0.1 mg/ml and used for subsequent experiments. Figure 1b demonstrated that the IC 50 value of Glu is 15.81 mM. Furthermore, Glu reduced cell viability in a concentration-dependent manner compared with the control group. At the concentrations of 5 mM, 10 mM, and 15 mM, the cell viability of Glu had no significant difference; while the cell viability of Glu was significantly decreased at the concentrations of 20 mM, 30 mM, and 40 mM, and the cell viability reduced to 46% following 20 mM Glu stimulation. Therefore, the damaging concentration of Glu was 20 mM and used for subsequent experiments.

### Effects of Moschus on Glu-induced PC12 cell injury

**Figure 2:**
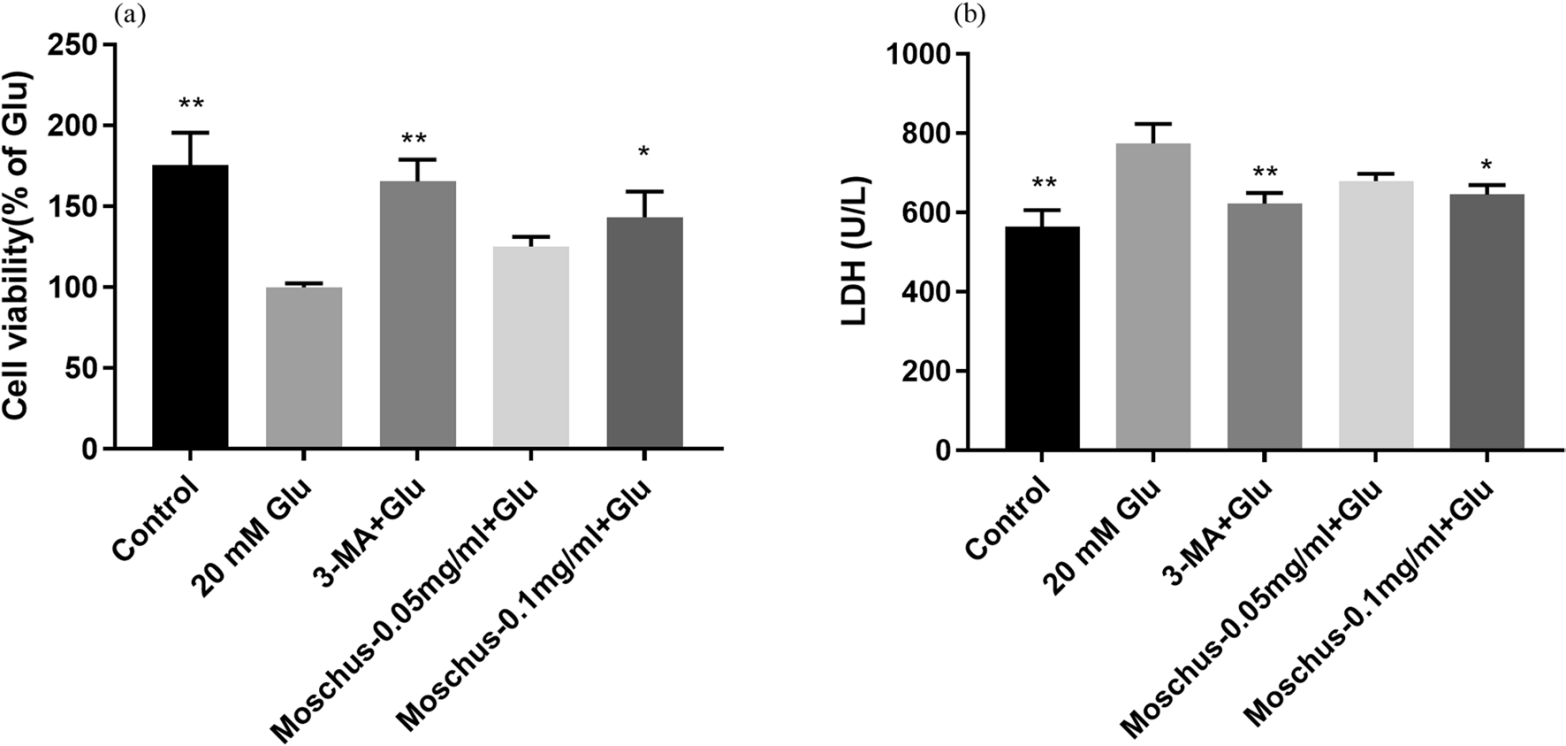
The effect of Moschus on cell viability and LDH of PC12 cells after Glu stimulation. (**a**) The effect of Moschus on Glu-induced cell viability in PC12 cells. (**b**) The effect of Moschus on Glu-induced LDH release in PC12 cells. *p < 0.05 and **p < 0.01 versus Glu-induced group.

Based on the screening drug concentrations, we evaluated whether Moschus possesses protective efficacy against Glu-induced cellular damage. Figure 2a indicated that the cell viability dramatically decreased after Glu stimulation compared with the control group, while the pretreatment of Moschus and 3-MA notably increased the cell viability. Figure 2b demonstrated that LDH release increased by 1.373-fold after Glu stimulation compared to the control group, while the pretreatment of Moschus and 3-MA notably reduced LDH release. These results showed that Moschus might be protective against Glu-induced cellular injury in PC12 cells.

### Effects of Moschus on Glu-induced PC12 cell apoptosis

**Figure 3:**
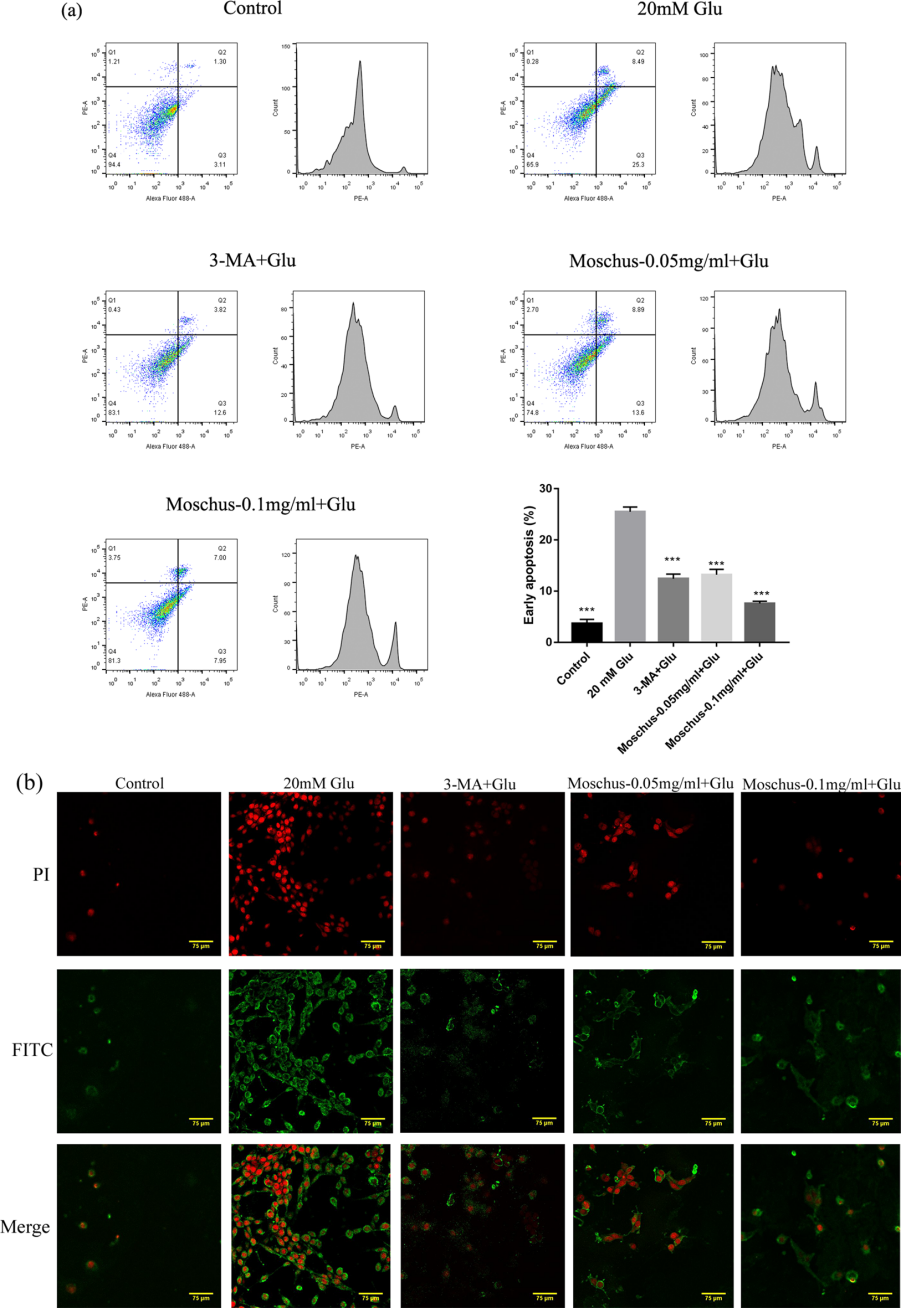
Effects of Moschus on Glu-induced cell apoptosis in PC12 cells. (**a**) Apoptosis was analyzed by flow cytometry using the Annexin V/PI double staining method. (**b**) Apoptosis was analyzed by laser confocal scanning microscopy using the Annexin V/PI double staining method. ***p < 0.001 versus the Glu-induced group.

Flow cytometry was used to measure the apoptotic rate of PC12 cells to explore whether Moschus affects Glu-induced cell apoptosis. Figure 3a indicated that the early apoptosis rate markedly increased after Glu stimulation compared with the control group, while the pretreatment of Moschus and 3-MA significantly decreased the early apoptosis rate. Figure 3b showed that weaker Annexin V fluorescence and PI fluorescence were exerted in the control group; compared with the control group, the most apparent Annexin V fluorescence and PI fluorescence were observed after Glu stimulation; while compared with the Glu group, Annexin V fluorescence and PI fluorescence were inhibited after the pretreatment of Moschus and 3-MA. These results indicated that Moschus might protect PC12 cells from Glu-induced cell apoptosis.

### Effects of Moschus on Glu-induced loss of membrane potential

**Figure 4:**
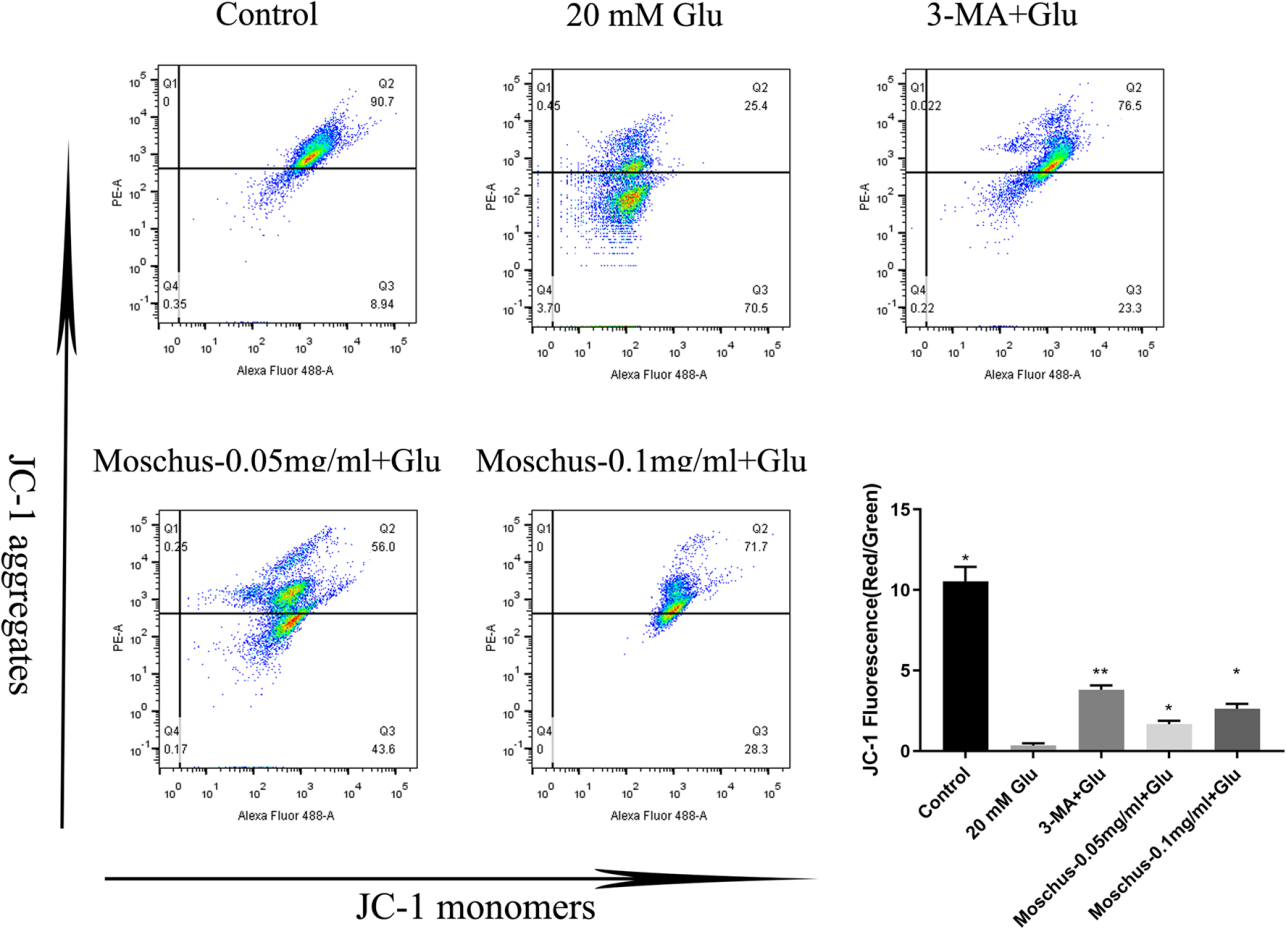
Effects of Moschus on Glu-induced loss of membrane potential in PC12 cells. *p < 0.05 and **p < 0.01 versus the Glu-induced group.

Mitochondrial damage is the main factor leading to apoptosis^109^. It is thought that apoptosis activation is preceded by loss of membrane potential in mitochondria^110^. The MMP is considered as an indicator of the state of the mitochondria^111^. A JC-1 mitochondrial membrane potential assay kit was used to measure the MMP. As shown in Fig. 4, compared with the control group, the ratio of red and green fluorescence drastically decreased after Glu-stimulated PC12 cells, while the percentage of red and green fluorescence significantly increased after the pretreatment of Moschus and 3-MA. Therefore, Moschus could attenuate Glu-induced loss of membrane potential in mitochondria in PC12 cells.

### Effects of Moschus on Glu-induced mitochondrial ROS generation

**Figure 5:**
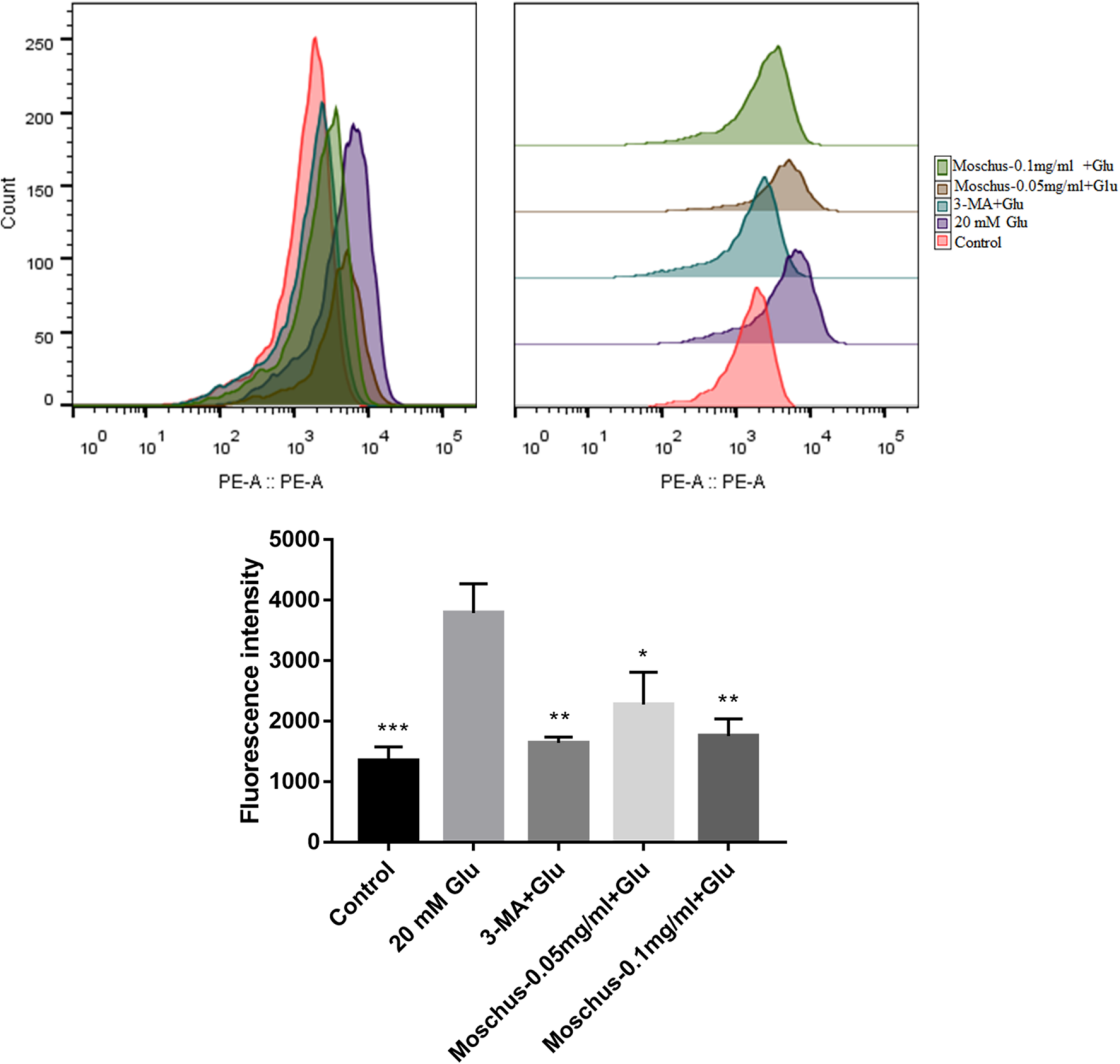
Effect of Moschus on Glu-induced mitochondrial ROS levels. *p < 0.05, **p < 0.01, and ***p < 0.001 versus Glu-induced group.

Damaged mitochondria generate excess ROS, leading to a reduction of MMP^112^. The MitoSOX Red was commonly applied to detect mitochondrial ROS levels. The result was shown in Fig. 5, the peak of mitochondrial ROS in Glu-stimulated PC12 cells was significantly moved to the right, indicating a significant increase in mitochondrial ROS generation, and adding Moschus and 3-MA incubation dramatically shifted the peak of mitochondrial ROS to the left, indicating a significant reduction in mitochondrial ROS generation compared with Glu-stimulated group. The result showed that Moschus protects PC12 cells against Glu-induced cell injury by inhibiting the accumulation of mitochondrial ROS.

### Effects of Moschus on Glu-induced cellular damage through regulating autophagy pathway

**Figure 6:**
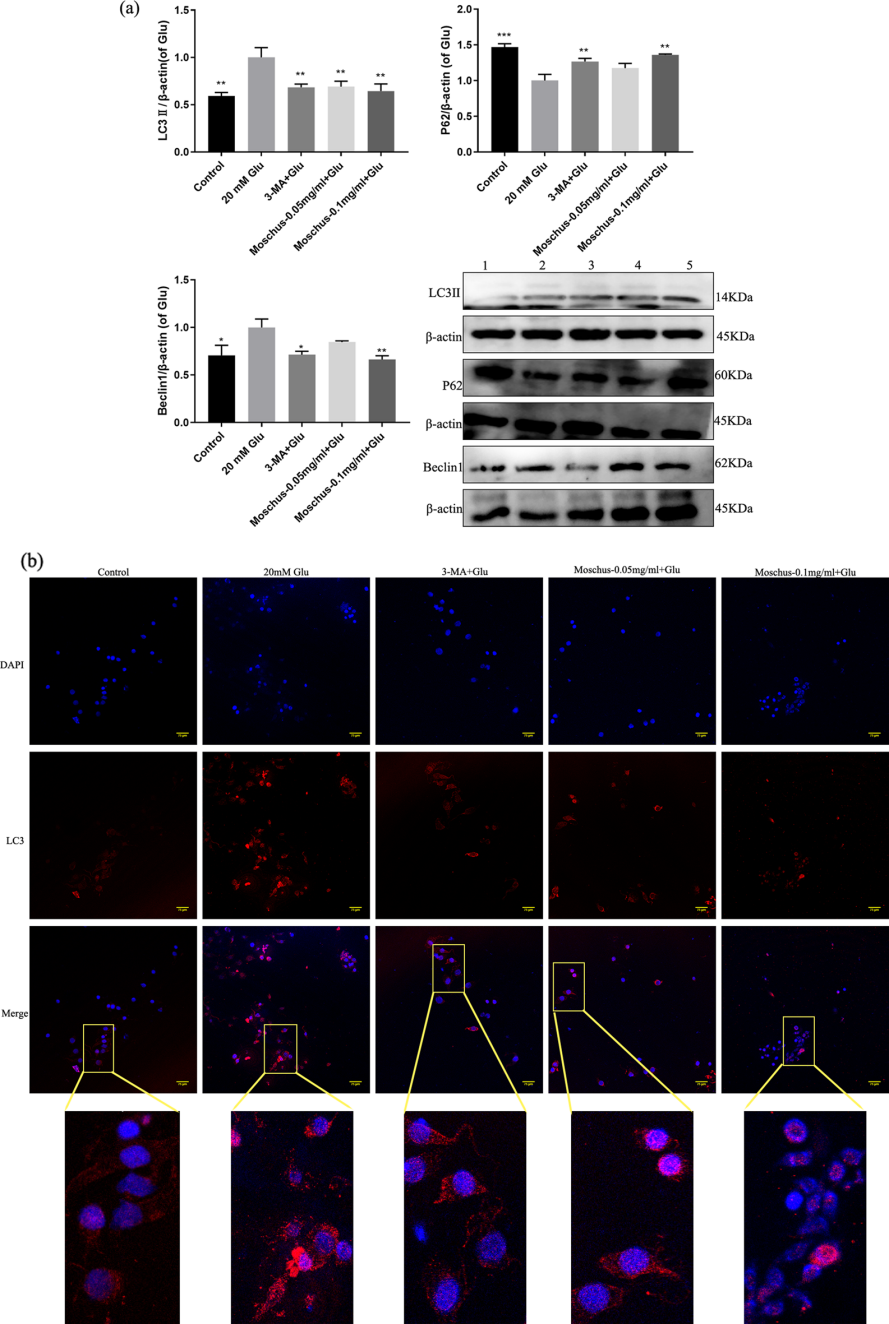

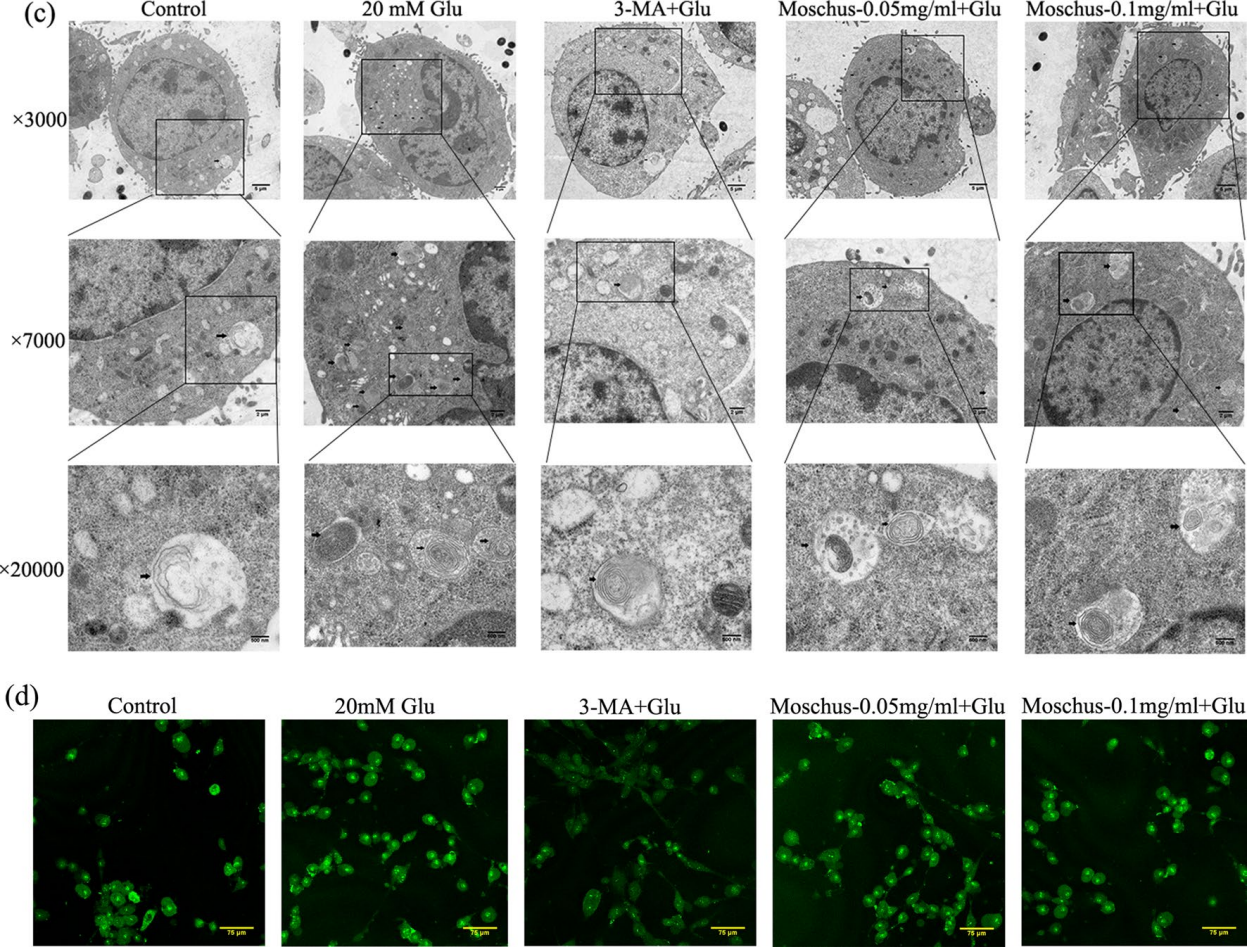
Effects of Moschus on autophagy-related proteins and Glu-induced autophagy. (**a**) Effects of Moschus on Glu-induced Bclin1, p62, and LC3 protein expression by western blot analysis. 1: Control; 2: 20 mM Glu; 3: 3-MA + Glu; 4: Moschus-0.05 mg/ml + Glu; 5: Moschus-0.1 mg/ml + Glu. (**b**) Immunofluorescence staining of LC3 was performed. (**c**) The autolysosomes or autophagosomes were monitored by TEM assay. Black arrows: autophagosomes/autolysosomes, scale bars: 5 μm at × 3000, 2 μm at ×7000, and 500 nm at ×20,000. (**d**) The fluorescent dyes of MDC detected the autophagic vacuole. *p < 0.05, **p < 0.01, and ***p < 0.001 versus the Glu-induced group.

Autophagy is the process of digesting cytoplasmic components by lysosomal degradation^113^. LC3, Beclin 1, and p62 are considered as indicators of autophagy^114^. As shown in Fig. 6a, Glu stimulation increased the Beclin 1 protein and LC3II protein expression but reduced the p62 protein expression, while the pretreatment of Moschus and 3-MA reduced the Beclin 1 and LC3II protein expression, increased the p62 protein expression. Furthermore, immunofluorescence was also applied to evaluate LC3 protein fluorescence. As shown in Fig. 6b, the result demonstrated that the number of LC3^+^ vesicles increased significantly in the Glu-stimulated group, while the number of LC3^+^ vesicles decreased after the pretreatment of 3-MA and Moschus. The mitochondrion is regarded as the bioenergetic center of cells, which is also vital to autophagy^115^.

The TEM assay was used to monitor cellular ultrastructure and autophagy. Figure 6c indicated that autolysosomes or autophagosomes markedly increased after Glu stimulation and mitochondria‐like structures could be observed in some autophagosomes; the pretreatment of Moschus and 3-MA notably decreased autolysosomes or autophagosomes. Meanwhile, the formation of double-membrane vesicles was regarded as the characterization of autophagy^116^. Additionally, the fluorescent dyes of MDC were used to detect the autophagic vacuoles. The MDC dye specifically accumulates in autophagic vacuoles, and the fluorescence intensity is positively related to autophagic vacuole number^117^. Figure 6d showed that Glu stimulation markedly increased intracellular MDC fluorescence compared to the control group. Compared with the Glu-stimulated PC12 cell model, intracellular MDC fluorescence reduced significantly after the pretreatment of 3-MA and Moschus. These results manifested that Moschus might ameliorate Glu-induced cell injury via regulating the autophagy pathway.

### Effects of Moschus on Glu-induced cellular damage through regulating apoptosis pathway

**Figure 7:**
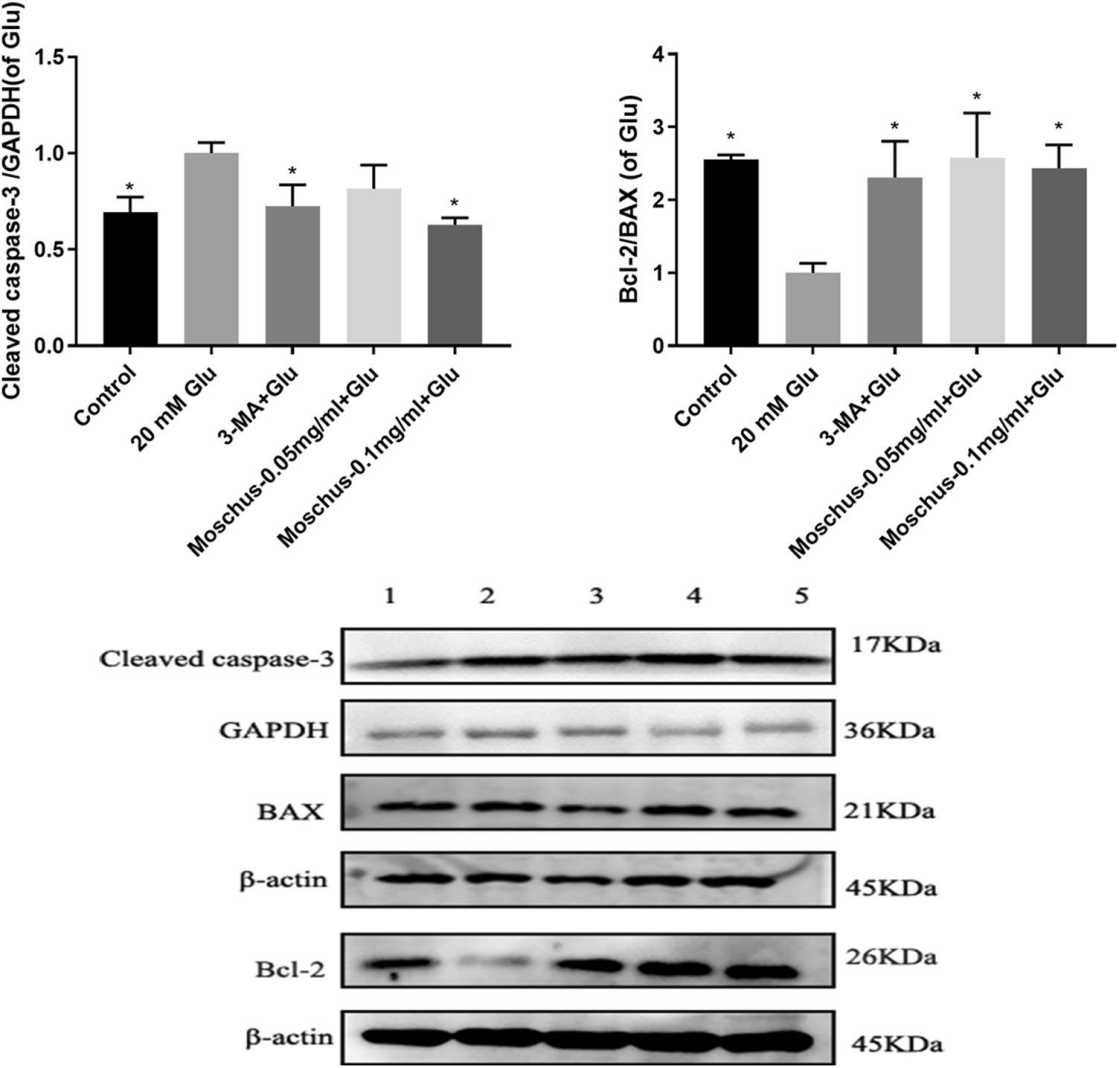
The effect of Moschus on apoptosis-related proteins. The cleaved caspase-3, BAX, and Bcl-2 protein expressions were measured by western blot analysis. 1: Control; 2: 20 mM Glu; 3: 3-MA + Glu; 4: Moschus-0.05 mg/ml + Glu; 5: Moschus-0.1 mg/ml + Glu. *p < 0.05 versus the Glu-induced group.

Studies have shown that Bcl-2 family members Bcl-2 and Bcl-xL are the main autophagy regulators^118,119^. The anti-apoptotic Bcl-2 family members have been identified to interact with Beclin 1 and reduce autophagy^120^. BAX is used as a pro-apoptotic protein to participate in apoptosis^121^. The cleaved caspase-3 is considered as an early apoptotic marker^122^. To explore whether the protective effect of Moschus is associated with the regulation of the apoptosis signaling pathway, the Bcl-2, BAX, and cleaved caspase-3 protein was investigated. As shown in Fig. 7, Glu stimulation increased the cleaved caspase-3 protein expression and decreased the ratio of Bcl-2/BAX, while the pretreatment of Moschus and 3-MA reduced the cleaved caspase-3 protein expression and increased the ratio of Bcl-2/ Bax in Glu-induced PC12 cell damage. The data demonstrated that Moschus might protect PC12 cells against Glu-induced injury through regulating the apoptosis pathway (Supplementary file 1).

## Discussion

AD is a common neurodegenerative disease characterized by declines in short-term memory and cognition^123^. Glu is involved in learning, memory, and synaptic plasticity^124,125^. Research has demonstrated that excitotoxicity caused by Glu is ubiquitous in AD^15^, such as neuronal death, oxidative stress, neuroinflammation, and synaptic loss. In clinical trials, compared to healthy controls, increased levels of Glu were observed in the cerebrospinal fluid (CSF) of patients with probable AD^126,127^. In comparison to patients with mild cognitive impairment, the Glu levels were also significantly elevated in the CSF of AD patients^128^. The Glu transporter function was decreased in the brain of AD patients, and the functional defect of Glu transporters might cause excessive Glu in the synaptic cleft and excitatory neuron damage^129^. In the animal experiment, elevated extracellular Glu levels were observed in APP/PS1 dentate gyrus, CA3, and CA1 hippocampal subregions from 6 months of age^130^. In the cellular experiment, cell apoptosis, ROS generation, and caspase-3 protein expression increased in Glu-induced PC12 cells^131^. It was found that Glu-induced cytotoxicity was associated with oxidative stress, mitochondrial imbalances, and autophagy imbalances^132^. Additionally, the apoptotic cell death caused by Glu toxicity was accelerated by mitochondrial dysfunction and ROS overproduction^133^. Therefore, Glu were selected as a damaging agent in this study.

The current evidence highlights decoupling in the mitochondrial respiratory chain^134^ and mitochondrial dysfunction stress are associated with Glu-mediated excitotoxicity^135^. Meanwhile, Glu-induced toxicity is regarded to induce mitochondrial dysfunction^136^ which is regarded as a critical early event required to induce apoptosis^137^. Mitochondria are involved in programmed cell death, especially apoptosis^138^ and ROS generation^139^. The mitochondria are taken as the focal point of cell apoptosis^140^. The loss of MMP is taken as a critical marker in apoptosis^141^. Cell apoptosis is the process of programmed cell death and plays a crucial role in regulating normal tissue homeostasis^142^. As a pro-apoptotic protein, BAX participates in apoptosis through the mitochondrial pathway^121^. The activation of BAX and the translocation from BAX to the mitochondria could cause mitochondrial dysfunction and apoptosis^143^. Meanwhile, the loss of MMP results in the activation of caspase cascades after releasing cytochrome c into the cytosol^144^. Bcl-2 family proteins are considered as the key regulators of mitochondrial outer membrane permeabilization^145^. Additionally, the excessive production of mitochondrial ROS leads to cell apoptosis^146^. In our study, the result indicated that Glu stimulation increased the loss of membrane potential, mitochondrial ROS production, and cleaved caspase-3 protein expression, while these results were reversed after the pretreatment of Moschus and 3-MA.

**Figure 8:**
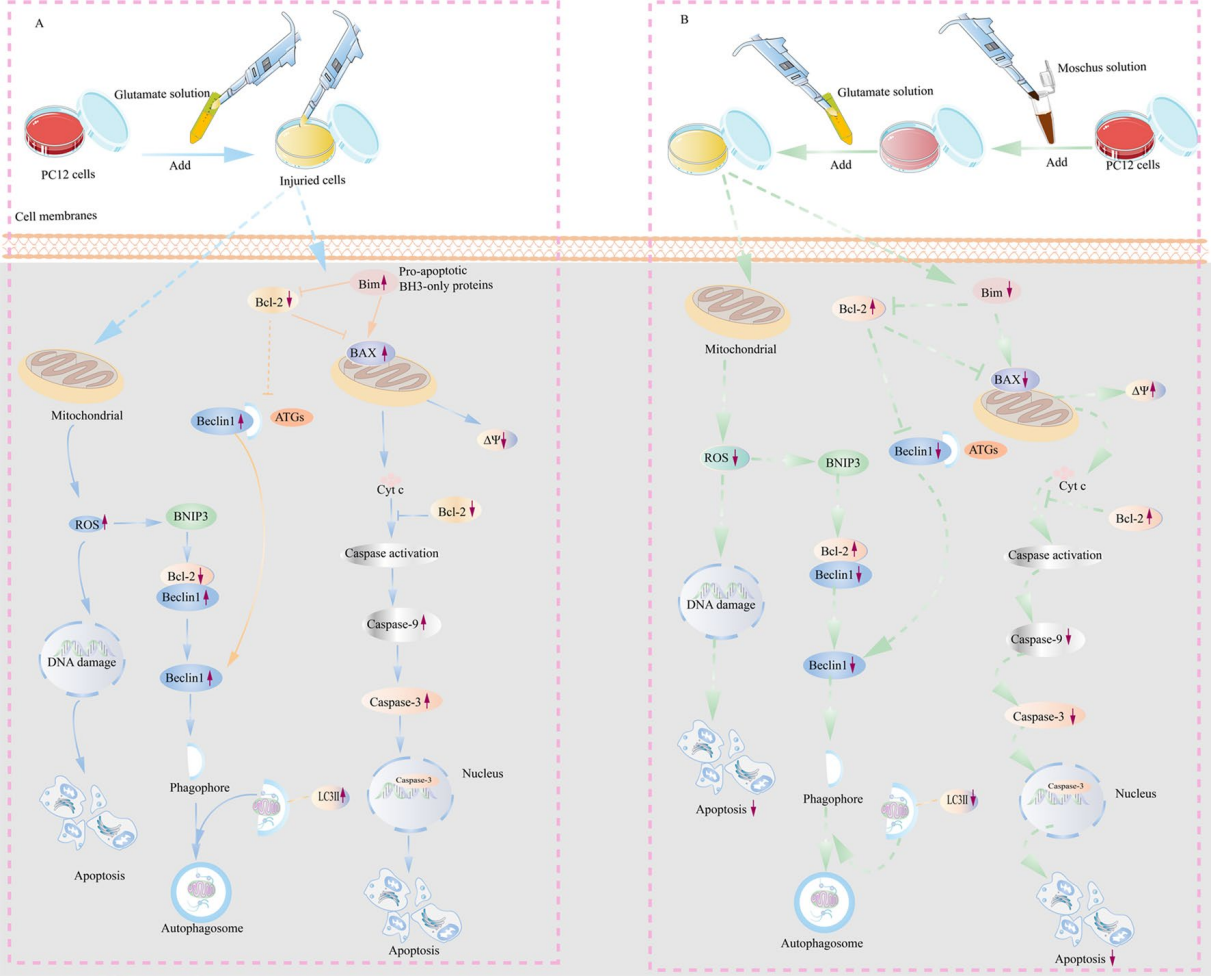
The potential mechanism between Glu and Moschus in PC12 cells. (**A**) The potential mechanism involved in Glu-induced injury. (**B**) The mechanisms involved in the protective effect of Moschus in Glu-induced injury.

Previous studies suggest that autophagy could participate in the pathogenesis of AD^147^. Various stress pathways could elicit autophagy and apoptosis; the process of autophagy is parallel to apoptosis^148^. Unlike apoptosis, autophagy provides energy and nutrients to promote cell survival by degrading cytoplasmic components in the lysosomes^149^. Beclin 1, as a Bcl-2-interacting protein, is essential for the induction of autophagy^150^. However, the Bcl-2 protein functions not only as an antiapoptotic protein but also as an anti-autophagy protein. Additionally, the interaction between the Bcl-2 protein and Beclin 1 protein could inhibit autophagy^119^. Furthermore, cleavage of autophagy proteins by caspases inhibits autophagy, especially p62^151^. The potential mechanism of the effect of Moschus and Glu in PC12 cells is showed in Fig. 8. We explored whether Moschus prevent Glu-induced cellular injury through the regulation of autophagy. The results demonstrated that Glu stimulation increased the Beclin 1 and LC3II protein expression, and reduced the p62 protein expression, while the pretreatment of Moschus and 3-MA reduced the Beclin 1 and LC3II protein expression, increased the p62 protein expression. The TEM assay and MDC assay showed that Glu stimulation might increase autophagic impairment, while the pretreatment of Moschus and 3-MA might reduce Glu-induced autophagic impairment.

Modern pharmacology suggests that Moschus possesses the effect of neuroprotective, anti-apoptotic, anti-oxidant, and immunity-enhancing biological activities^152^, and is broadly used in the central nervous system, cardiovascular and cerebrovascular systems. The protective effect of Moschus on Glu-induced cellular damage was investigated. 17 chemical compounds from the Moschus sample were identified through GC–MS analysis in our study. Muscone, Prasterone-3-sulfate, Cholesta-3,5-diene, 3α-hydroxy-5β-androstan-17-one, and Androstane-3,17-dione, (5β)- possess protective effects on CNS. The neurological scores and infarct volumes in transient middle cerebral artery occlusion rats could be decreased after treating with muscone by vein injection (2.67 mg/kg or 5.33 mg/kg)^153^. Muscone dramatically alleviated Glu-induced apoptosis and oxidative stress in PC12 cells^154^. Prasterone-3-sulfate, also called DHEAS, is a naturally occurring androstane steroid^155–157^. Acetylcholine released from hippocampal neurons is increased by intraperitoneal injection of 25–250 µmol/kg DHEAS in anesthetized rats^158^. Furthermore, learning impairment in the water-maze test and spontaneous alternation deficits in the Y-maze test was attenuated by subcutaneous injection of 20 mg/kg DHEAS in the scopolamine-induced mice model^159^. All cholinergic neurons employ the neurotransmitter Ach^160^, which is highly related to learning and memory functions^161^. Furthermore, the degeneration of cholinergic neurons is a prominent feature of AD^162^. Additionally, the increased release of vascular endothelial growth factor (VEGF) from natural killer (NK) cells co-incubated with DHEAS was found in patients with vascular dementia (VAD) and AD, while NK cells co-incubated with Aβ_1-42_ entirely inhibited the VEGF release in patients with VAD and AD^163^. Reduced levels of VEGF, the prototype angiogenic factor, may contribute to the neurodegenerative process and vascular dysfunction in AD^164^. The cognitive impairment, Aβ burden, and hyperphosphorylated-tau level were ameliorated after implanting encapsulated VEGF-secreting cells in APP/PS1 mice^165^. Cholesta-3,5-diene, as a cholesterol metabolite^166^, is considered an oxysterol that could regulate cellular cholesterol homeostasis^167^. Oxysterol possessed biological effects including protein prenylation, apoptosis, modulation of sphingolipid metabolism, and platelet aggregation^168^. 3α-hydroxy-5β-androstan-17-one, also called etiocholanolone, is an etiocholane (5β-androstane) and an endogenous 17-ketosteroid produced by testosterone metabolism^169^, and is also known to be an inhibitory androstane neurosteroid^170^. Testosterone exhibits a neuroprotective effect by reducing Aβ production, improving synaptic signaling, and combating neuronal death^171^. Androstane-3,17-dione, (5β)-, also known as Etiocholanedione, is a naturally occurring etiocholane (5β-androstane) steroid and an endogenous metabolite of androgens and has the anti-inflammatory action^172^. Therefore, it is reasonable to speculate that the neuroprotective effect of Moschus may be attributed to the synergistic effects of these monomers. Muscone, Prasterone-3-sulfate, Cholesta-3,5-diene, 3α-hydroxy-5β-androstan-17-one, and Androstane-3,17-dione, (5β)- might be potential candidates for developing central nervous system drugs, especially anti-AD drugs.

In summary, the study manifested that Moschus might possess the neuroprotective effect in Glu-induced neurotoxicity. In-depth studies on the multiple components of Moschus might provide a potential target for preventing and treating AD.

### Supplementary Information

Supplementary Information.

## Data Availability

The data used to support the findings of this study are available from the corresponding authors upon request.

## Abbreviations

AD: Alzheimer's disease
Glu: Glutamate
3-MA: 3-Methyladenine
Ach: Acetylcholine
Trk: Tyrosine kinase
p75^NTR^: P75 neurotrophin receptor
BCF: Basal cholinergic forebrain
ROS: Reactive oxygen species
Aβ: Amyloid-β
Bcl-2: B-cell lymphoma 2
BBB: Blood–brain barrier
EB: Evans blue
CIRI: Cerebral ischemia–reperfusion injury
H_2_O_2_: Hydrogen peroxide
NGF: Nerve growth factor
ANP: Angong Niuhuang Pill
THD: Tongqiao Huoxue Decoction
XNJI: Xingnaojing Injection
CCK-8: Cell counting kit-8
PCD: Programmed cell death
MDC: Dansylcadaverine
LDH: Dehydrogenase
BCA: Bicinchoninic acid
CSF: Cerebrospinal fluid
PS: Phosphatidylserine
MMP: Mitochondrial membrane potential
TEM: Transmission electron microscope
VEGF: Vascular endothelial growth factor
NK: Natural killer
VAD: Vascular type
