# Supplementary material for: Moschus ameliorates glutamate-induced cellular damage by regulating autophagy and apoptosis pathway
Source: Sci Rep. 2023 Oct 30;13:18586. doi: 10.1038/s41598-023-45878-7 (PMC10616123; doi:10.1038/s41598-023-45878-7)
Supplement: Supplementary file 1 — Supplementary Information. [file 41598_2023_45878_MOESM1_ESM.pdf]

<table>
<tr><td>Bcl2</td><td></td><td></td></tr>
<tr>
<td>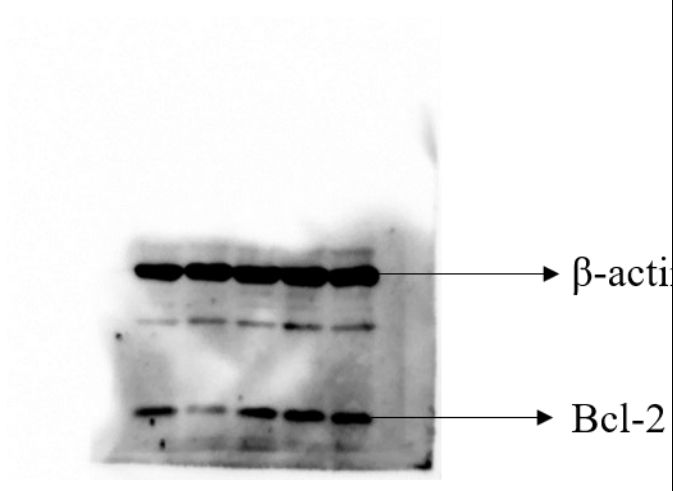
</td>
<td>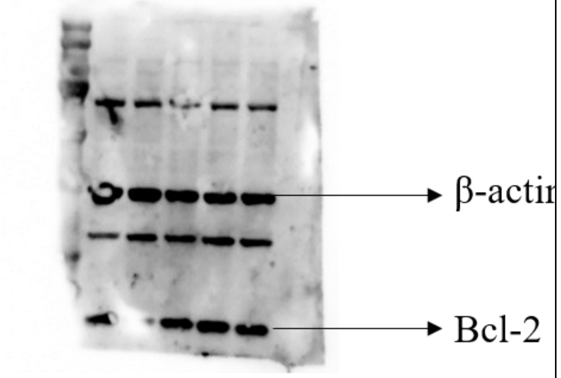
</td>
<td>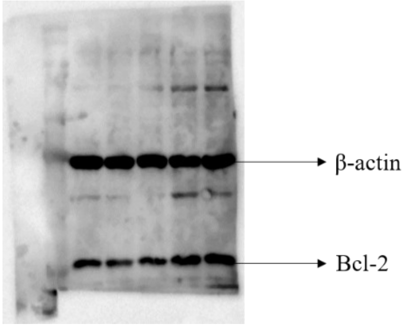
</td>
</tr>
<tr><td>BAX</td><td></td><td></td></tr>
<tr>
<td>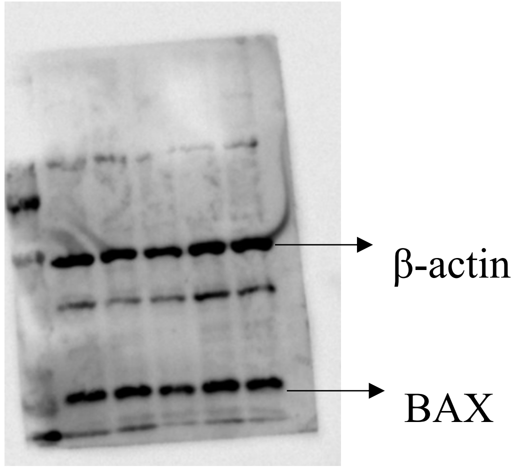
</td>
<td>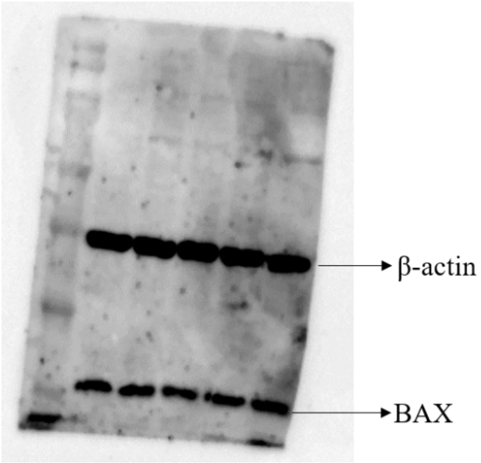
</td>
<td>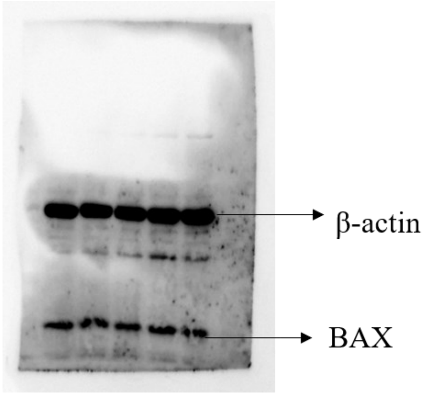
</td>
</tr>
</table>

<table>
<tr><td>Cleaved capase-3</td><td></td><td></td></tr>
<tr><td>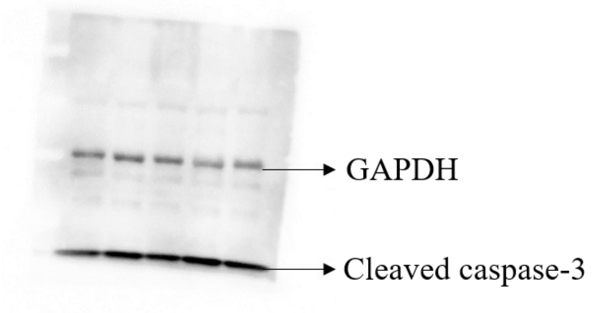
</td><td>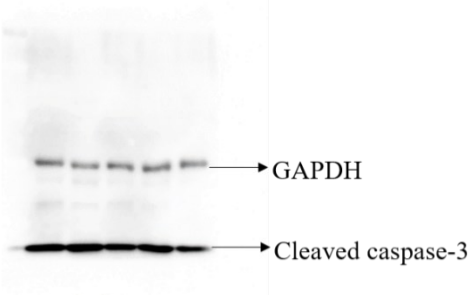
</td><td>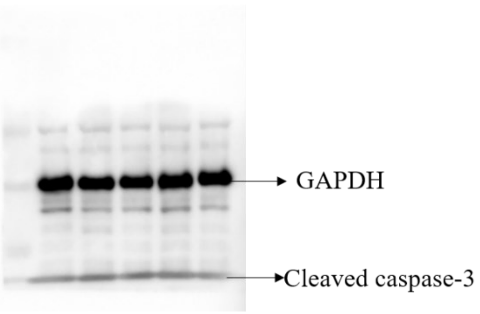
</td></tr>
<tr><td>Beclin1</td><td></td><td></td></tr>
<tr><td>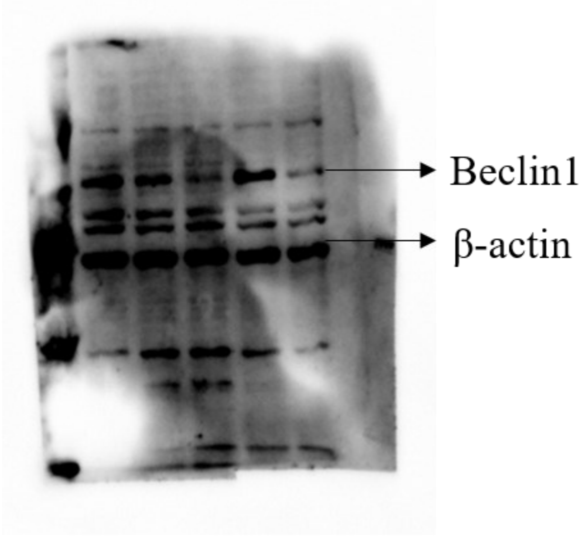
</td><td>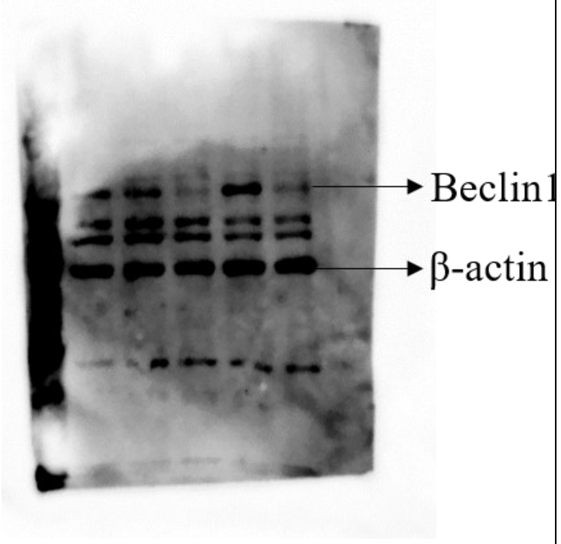
</td><td>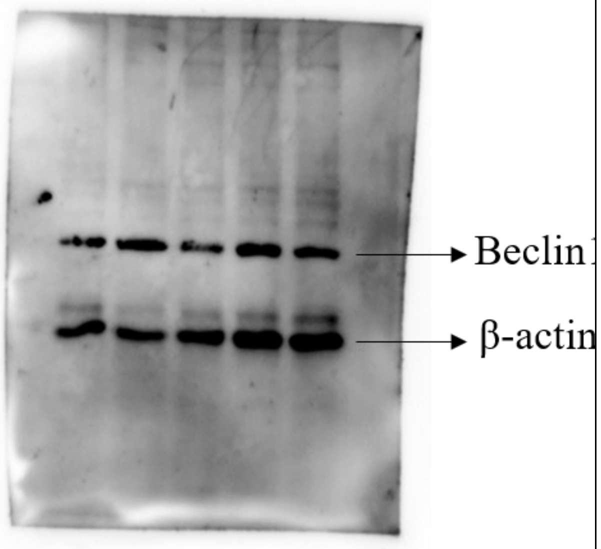
</td></tr>
<tr><td>P62</td><td></td><td></td></tr>
</table>

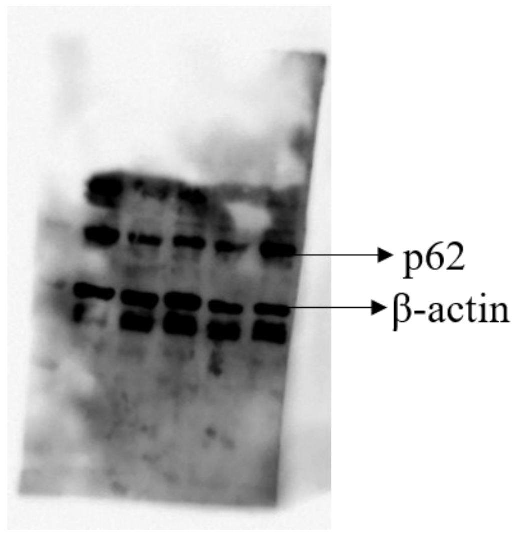	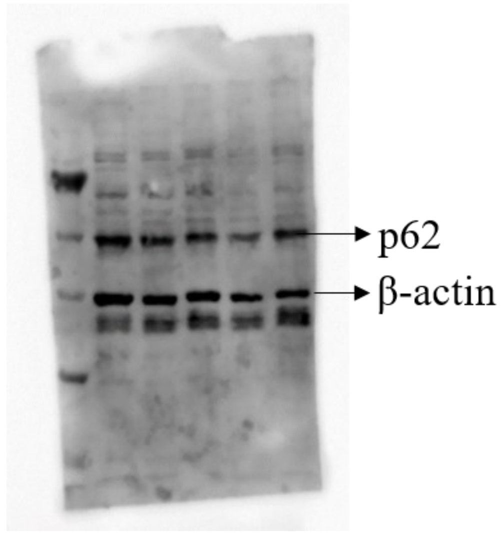	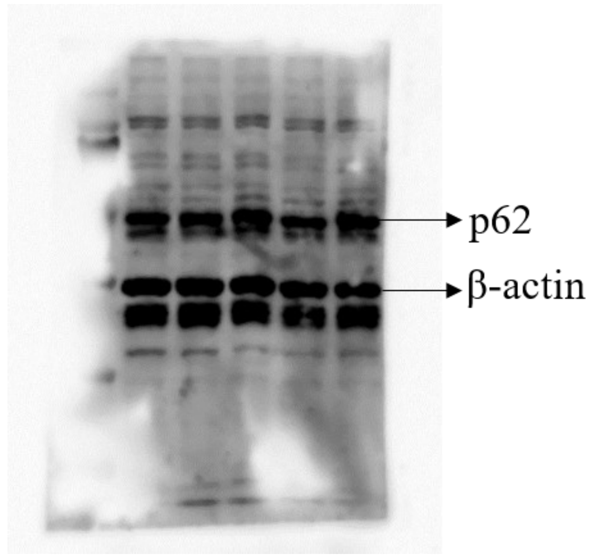
LC3		

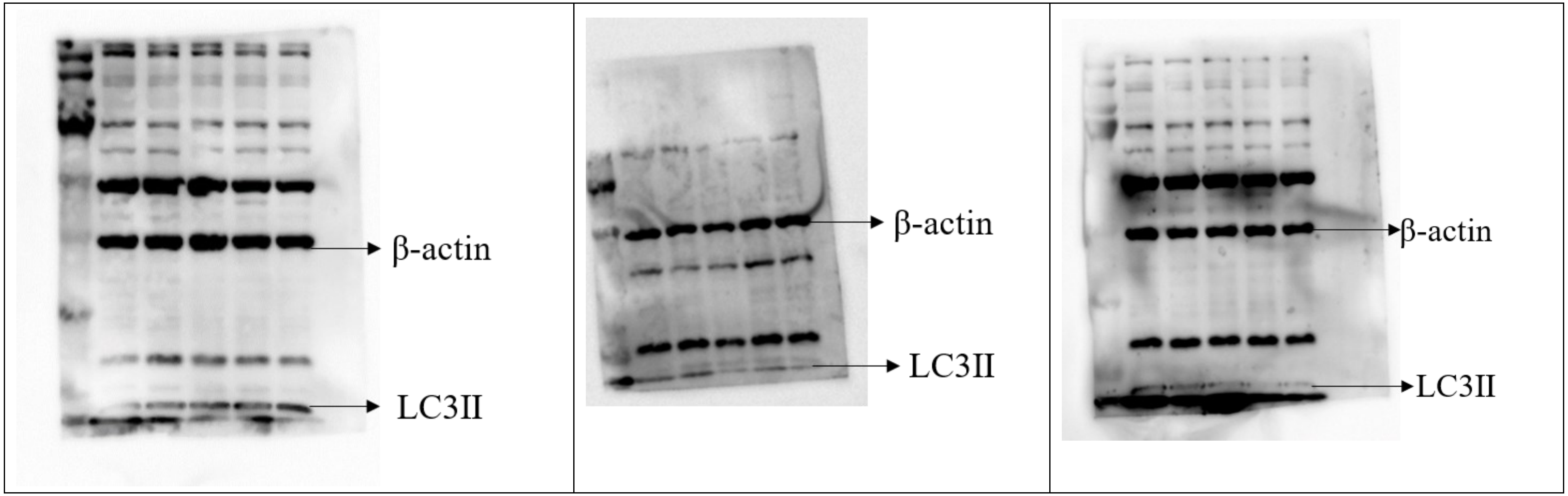
β-actin
LC3II
β-actin
LC3II
β-actin
LC3II
